# Early-life adversities and later-life reproductive patterns in women with fully traced reproductive history

**DOI:** 10.1038/s41598-023-36226-w

**Published:** 2023-06-08

**Authors:** Magdalena Klimek, Sonja Entringer, Agnieszka Matras, Mateusz Blukacz, Ilona Nenko, Andrzej Galbarczyk, Grazyna Jasienska

**Affiliations:** 1 Department of Environmental Health, Faculty of Health Sciences, Jagiellonian University Medical College, Krakow, Poland; 2 Institute of Medical Psychology, Charité - Universitätsmedizin Berlin, Corporate Member of Freie Universität Berlin and Humboldt-Universität Zu Berlin, Berlin, Germany; 3 Department of Pediatrics, University of California Irvine, Irvine, CA USA; 4 Institute of Psychology, Faculty of Social Sciences, University of Silesia, Katowice, Poland

**Keywords:** Developmental biology, Evolution, Risk factors

## Abstract

One of the most crucial determinants of early-life development is the experience of childhood adversities. However, limited evidence is available for how these experiences shape later-life reproductive outcomes in women. Here we test the association between early-life adversities and reproductive parameters in women. Post-reproductive women (N = 105; mean age = 59.7; SD = 10.09) were recruited at the Mogielica Human Ecology Study Site in Poland within a traditional population with a low prevalence of birth control usage and fully traced reproductive histories. Reproductive parameters, as well as exposure to early-life abuse and neglect, were assessed using questionnaires. Childhood adversity was associated negatively with age at menarche (*p* = 0.009). Analyses on specific subtypes revealed that compared to women who did not experience any kind of early-life adversities in childhood, those who were exposed to emotional (*p* = 0.007) and physical (*p* = 0.023) neglect had an earlier age at menarche, those who experienced emotional abuse reported an earlier age at first birth (*p* = 0.035), and those who were exposed to physical abuse gave birth to fewer number of sons (*p* = 0.010). Our results suggest that women exposed to childhood adversities experience earlier physiological reproductive readiness and timing of the first birth, but their overall biological condition might be impaired as they bear fewer sons.

## Introduction

Life history theory provides a theoretical framework into understanding the early-life embeddings of later-life reproductive patterns. Life history strategy is influenced by the environmental conditions experienced by an individual, mainly during the early years of development. Under challenging circumstances, especially those threatening future survival (e.g., food deprivation, high population mortality rate), a faster life history strategy is often adopted and manifested by higher investment in the current reproduction rather than the future one, which might never occur^1,2^. In stressful and unpredictable environments, the allocation of resources for physiological reproductive readiness rather than for the growth or maintenance of the organism is expected^3,4^. On the one hand, an earlier start of reproductive life gives a potential to produce a large number of offspring among whom at least some are likely to survive, but on the other hand, such accelerated reproductive pace might lead to reduced investment in the offspring and compromise their survival^4,5^. Going further, as the Trivers-Willard hypothesis^6^ predicts, it should be beneficial for a mother to adjust her offspring’s sex ratio to the prevailing conditions and towards, more reproductively valued in harsh settings, daughters^7^ (but see:^8^). Yet, currently, the “fast-slow” paradigm^9^ faces a critique regarding the lack of empirical evidence underlying this phenomenon^10^.

Developmental conditions during earlylife may shape reproductive strategy, and there is evidence for the importance of the prenatal phase of life in influencing later-life reproductive traits^11–14^. The window of developmental plasticity extends to childhood. One of the most crucial factors defining the quality of childhood environment is the experience of adversities such as psychological stress or traumas, poverty, abuse, or neglect. Early-life adversity not only profoundly increases the risk for psychiatric disorders (e.g., higher risk of depression or suicidal thoughts)^15,16^, but also for other chronic health problems, including a higher risk of noncommunicable diseases (e.g., cardiovascular diseases, diabetes, cancer)^17–19^, unhealthy behaviors (e.g., substance abuse or risky sexual activity)^20,21^, poor self-rated health^22^, higher overall mortality rate^23^ and faster cellular aging^24^. However, in humans there is limited evidence how these adversities shape later-life reproductive outcomes in women. Studies in animal models show mixed conclusions. While some suggested that stress exposure during early-life (e.g., separation from the mother) leads to delayed puberty and enhanced sexual motivation in rats^25^, and delayed sexual maturation in mice^26^, others do not support these findings^27^. In humans, results of studies that have linked female reproductive characteristics with early-life adversities suggest that women exposed to more adversity during childhood, including low socioeconomic status, exhibit earlier pubertal timing, an earlier start of reproductive life, increased sexual desires and more risky sexual behaviors^28,29^. However, some studies reported contradictory results, e.g., delayed puberty of women subjected to childhood stressors^30^. Also, a recent metanalysis on the association between early-life adversities and pubertal timing indicated a lack of such relationship when the total score of stressful exposures was investigated^31^.

So far, little is known about the potential impact of early-life stress and adverse conditions on female reproductive success (i.e., the number of children who were born alive and survived), sex ratio of children, or the course of lifetime reproductive history, particularly in post-reproductive women with low access to birth control and with full reproductive history traced. A study on Finnish historical data, covering the 1934–1944 period, has shown that girls who were evacuated from the city during the World War II (thus, potentially exposed to a higher level of psychological stress) gave birth to a larger number of children later in life (however, the effect was no longer observed after adjusting for the year of birth) comparing to the group without separation experience^32^. In a study based on historical data collected within the Newcastle Thousand Families Study, Sheppard and colleagues^29^ found an association between lower childhood socioeconomic status and poorer housing conditions, and earlier start of reproductive life and higher number of surviving children (but this association was fully mediated by the age at first reproduction). Those studies, however, applied an indirect measure of early-life stressful exposures related to e.g., political situation or economic position of a family, with no self-reported levels of adversities by the surveyed participants. In studies embedded in contemporary populations e.g., Yuan and colleagues^33^ found in Chinese middle-aged women (40 years and older) that those exposed to early-life threats experienced accelerated puberty, earlier age at first birth and increased total number of children born. However, the reproductive patterns of those women could be biased due to family planning policies introduced and implemented in China between 1980–2015 in order to limit the size of the population^34^.

The present study aimed to explore associations between self-reported childhood adversities (particularly emotional and physical abuse or neglect) and female reproductive characteristics. To the best of our knowledge, this is the first study surveying the link between early-life adversities assessed individually for each woman and her fully traced reproductive history in a population with highly limited usage of birth control. Based on the life-history evolutionary framework we predict that the exposure to higher levels of childhood adversity will result in a faster reproductive pace in women (earlier age at menarche, earlier age at first and last birth, shorter interbirth intervals, and a higher number of children born). As faster life history in women experiencing more early-life adversities might result in reduced investment per-offspring, we predict that those women will give birth to a relatively lower number of sons than daughters.

## Results

### CTQ summary score

In the first step, multiple general linear models (GLM) were conducted to test the relationship between the “CTQ sum” variable (sum of childhood adversity exposure across the four tested categories—emotional neglect, physical neglect, emotional abuse, and physical abuse) and the reproductive history parameters (Table 1). “CTQ sum” was negatively associated with age at menarche (*p* = 0.009), after adjusting for year of birth and number of years of education, suggesting that higher levels of childhood trauma exposure were linked to earlier age at menarche. No other statistically significant relationships between the “CTQ sum” score and any of the reproductive history parameters were observed (Table 1). In the second step, the four binary variables for each category of childhood adversity (emotional or physical abuse, emotional or physical neglect) were used as predictors in the analyses (Table 2). The binary variables were created as follows: Participants were assigned “0” if there was no experience of adversity at all (chosen answer “never true”); they were assigned “1” if they reported at least some adversities in any of the categories (chosen answers “rarely true”, “sometimes true”, “often true”, “very often true”).

**Table 1:** The relationship between “CTQ sum” and parameters of reproductive history.

	“CTQ sum”			
	Estimate	SE	t/z value	p
Age at menarche (years)^a,b^	− 0.05	0.02	− 2.68	**0.009**
Age at first reproduction (years)^a,b,c^	− 0.03	0.03	− 1.07	0.29
Age at last reproduction (years)^a,b,d^	0.07	0.06	1.10	0.28
Number of children born^a,b,d^	− 0.0009	0.007	†− 0.14	0.89
Proportion of sons to daughters^a,b^	− 0.0010	0.003	− 0.30	0.77
Mean inter-birth interval (months)^a,b,d^	0.11	0.25	0.45	0.65

^†^ Poisson distribution (z value).

Covariates: a—year of birth, b—number of years of education, c—age at marriage, d—age at first birth.

Significant values are in bold

**Table 2:** The difference between women exposed and non-exposed to each type of adversity in parameters of reproductive history.

	Emotional neglect			Physical neglect			Emotional abuse			Physical abuse		
	F	η_p_^2^	p	F	η_p_^2^	p	F	η_p_^2^	p	F	η_p_^2^	p
Age at menarche (years)^a,b^	7.52	0.072	**0.007**	5.33	0.052	**0.023**	1.73	0.018	0.19	0.51	0.005	0.48
Age at first reproduction (years)^a,b,c^	1.60	0.016	0.21	0.82	0.009	0.37	4.57	0.045	**0.035**	0.02	0.001	0.89
Age at last reproduction (years)^a,b,d^	0.08	0.001	0.78	0.15	0.002	0.70	0.40	0.004	0.53	0.07	0.001	0.78
Number of children born^a,b,d^	0.001	0.001	0.98	0.006	0.001	0.94	0.87	0.009	0.35	0.13	0.001	0.72
Proportion of sons to daughters^a,b^	2.01	0.021	0.15	0.25	0.003	0.62	0.47	0.005	0.50	6.94	0.067	**0.010**
Mean inter-birth interval (months)^a,b,d^	0.87	0.009	0.35	0.12	0.001	0.73	0.90	0.010	0.35	0.43	0.005	5.15

η_p_^2^—partial eta squared.

Covariates: a—year of birth, b—number of years of education, c—age at marriage, d—age at first birth.

Significant values are in bold

### Emotional neglect

**Figure 1:**
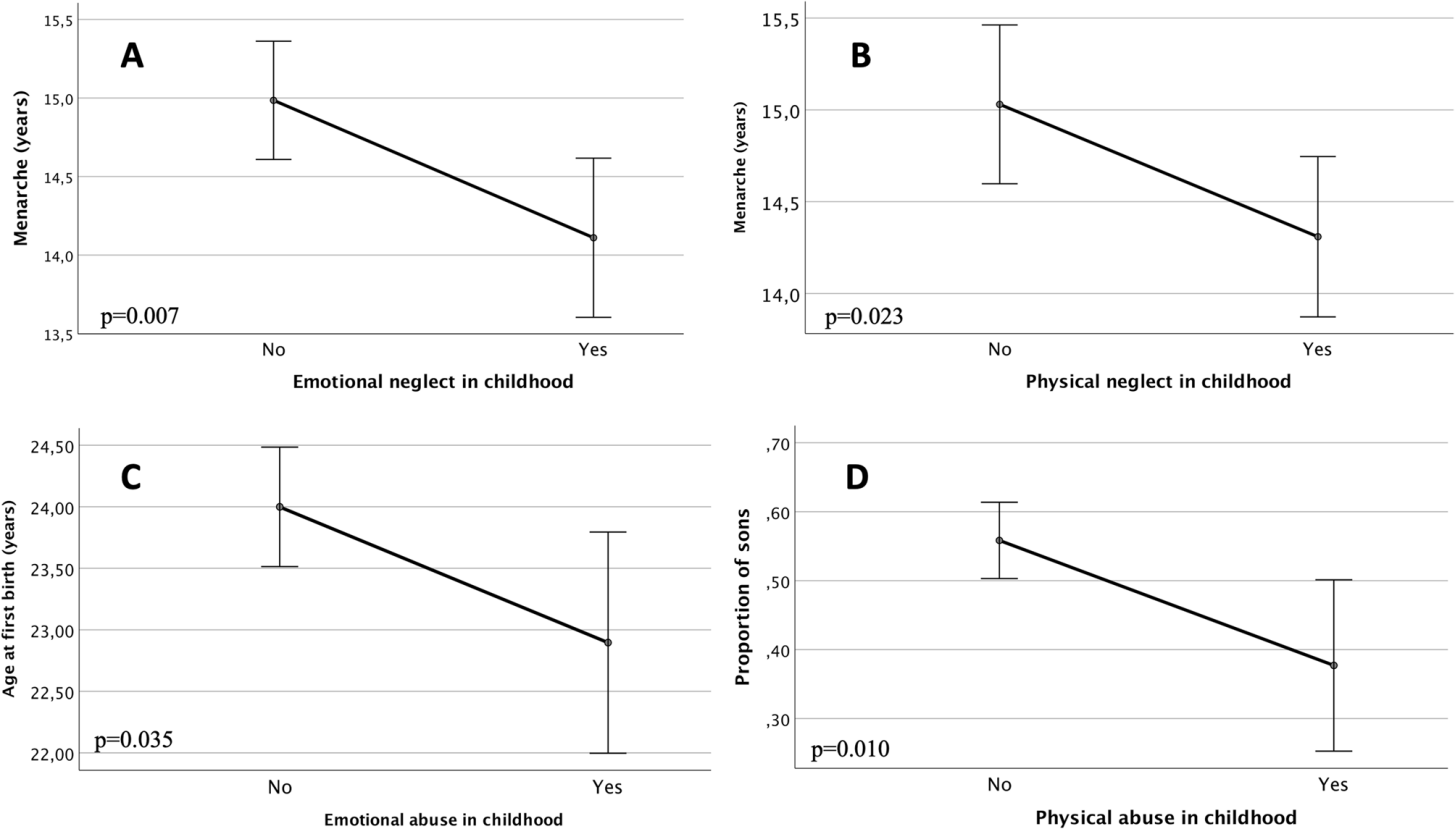
The association between different components of childhood stress and reproductive parameters: menarche (**A**, **B**), age at first birth (**C**), and proportion of sons (**D**). Lines and bars represent standard error of the mean (SEM).

Women who experienced emotional neglect during childhood had an earlier age at menarche than women who were not emotionally neglected, after adjusting for women’s year of birth and education (14.11 vs 14.99 years respectively; *p* = 0.007). On average, women exposed to emotional neglect had their first menstruation 10.56 months earlier compared to women who were not emotionally neglected (Fig. 1a).

### Physical neglect

Physical neglect in childhood was also associated with an earlier age at menarche, after adjusting for women’s year of birth and education (14.31 vs 15.03 years respectively; *p* = 0.023) (Fig. 1b). Exposure to physical neglect in childhood was associated with an earlier age at menarche that was on average reduced by 8.64 months.

### Emotional abuse

Emotional abuse was associated with earlier age at first birth, adjusting for women’s year of birth, education and age at marriage (22.90 vs 23.99, respectively; *p* = 0.035) (Fig. 1c).

### Physical abuse

Women who experienced physical abuse had a lower number of sons (expressed as the proportion of sons) than women who were not physically abused, after including women’s year of birth and education as covariates (0.38 vs 0.56 respectively; *p* = 0.010) (Fig. 1d). This corresponds to 18% decrease in the proportion of sons among women who were physically abused compared to those who were not.

All statistically significant effects were of medium sizes [partial eta squared (η_p_^2^) ranged from 0.045 to 0.072]. No associations between subscales of the CTQ and any of the other investigated reproductive parameters were observed (Table 2).

## Discussion

Results of this study suggest that childhood adversities have long-term impact on women’s reproduction. Women exposed to childhood adversities in general, but also to specific types of maltreatment, namely emotional and physical neglect, had an earlier age at menarche, those who experienced emotional abuse reported an earlier age at first birth, and those who were exposed to physical abuse had a lower proportion of sons. The results partially confirm our hypothesis of faster life history strategy and potentially lower biological condition of women subjected to early-life adversities. Although several explorations of this hypothesis were published^35,36^, to the best of our knowledge, this is the first study investigating this phenomenon in such a broad reproductive context and across the women’s lifespan, starting from the onset of puberty and ending at the age at last birth. Also, this study provides a novel perspective based on subjective assessment of early-life adversities among rural women with non-western lifestyles, with a very low prevalence of birth control methods usage. The need of surveying such populations was recently called to attention by others^10^.

A recent metaanalysis on childhood adversities and pubertal timing^31^ did not find statistically significant associations when the total score of adverse childhood experiences was explored, but associations were significant when specific types of childhood adversities were separately analyzed, namely the father's absence, family dysfunction, and sexual abuse. Apart from differences in demographic, social, and reproductive backgrounds of the women involved in studies included in this metaanalysis, one reason for inconclusive results between the studies involved in the metaanalysis may be that varying approaches were used for the assessment of early-life adversities. Our study comprehensively assessed general childhood trauma exposure, focusing on various aspects of adversities, i.e., emotional and physical abuse, and emotional and physical neglect. Considering the rather small to moderate overlap between those subcategories in this sample (correlation coefficients between each category ranged from 0.18 to 0.64), such an approach was justified and unveiled the effects which otherwise might have been blurred in a larger context of “general childhood adversities” used in preceding studies (for a review see^31^).

We have observed an earlier age at menarche in women with higher scores of overall childhood adversity exposure (“CTQ sum”), as well as in groups exposed to both emotional and physical neglect, compared to those non-exposed. Several potential mechanisms of the observed association seem plausible. While genetic factors are clearly influencing the onset of puberty^37^, the environmental causes are important as well, including increased levels of psychological stress resulting from various types of adversities. The potential pathway linking early-life stress exposure and onset of puberty could be alterations in the activity of the hypothalamic–pituitary–adrenal (HPA) axis and cortisol secretion. The HPA axis acts as an inhibitor of the hypothalamic-pituitary–gonadal (HPG) axis which is involved in initiation of puberty and reproduction^38,39^, proposed as “stress-suppression theory”^40^. However, contrary to this idea, previous prospective studies suggested that childhood stress of a chronic nature might result in downregulated HPA axis activity and attenuated cortisol levels, thus mitigate the suppression of the HPG axis and, consequently, accelerate the puberty^38,41^. Additionally, it has been suggested that early-life neglect could predispose children to excess body weight as an effect of a stress coping strategy^42,43^. Higher body fat may lead to higher sex hormone concentrations (i.e., estradiol) and therefore potentially contribute to the earlier onset of puberty in girls^44^. An additional potential mechanism which could contribute to the decreasing age at menarche in girls exposed to childhood adversities is an accelerated pace of aging, as evidenced by markers of cellular aging like telomere length or an aging-related DNA-methylation profile (e.g.,^45^). Early-life threats might lead to accelerated biological aging which might then act as a signal for the reproductive tract to hasten the pubertal timing (for a metanalysis see:^46^).

Our findings suggest an earlier age at first birth in women exposed to emotional abuse compared to women who were not emotionally abused. The earlier start of reproductive life, could also be mediated by the HPA and HPG axes interplay^38^, similarly to the earlier onset of menstruation. An alternative mechanism is also plausible. Early childhood is a phase of building secure attachments, usually with parents (or at least one of them). Children with secure emotional bonds to their caregivers that are surrounded by a safe, supporting, and comforting environment should adapt a slower life history strategy^47^ and delay their reproduction. Our result of a faster reproductive start in emotionally abused women is in accordance with this theory and is in line with a previously reported finding that women with more insecure attachment to their parents during childhood and adolescence have a lower age at first birth^48^.

The experience of physical abuse could result in worsened physical health both during childhood^49^ and adulthood^50^. Therefore, the observed lower proportion of sons in women exposed to physical abuse might emerge from their poorer biological condition, as male offspring require higher energetic investment both during pregnancy^51^ and lactation^52^. Also, this result lies in accordance with the Trivers-Willard hypothesis which predicts that females of poor biological condition and originating from harsh environments will adjust their sex ratio and invest in conceiving daughters, who, subsequently, even in poorer condition, will out-reproduce sons, a strategy resulting in a higher number of grandchildren for a woman^6^.

We did not observe any associations between childhood adversities and traits related to the pace of reproductive span—inter-birth intervals, number of children, and age at last birth. A lack of such relationship seems to be supported by the fact that women exposed to psychological stress during early years of life have a higher ovarian reserve (reflected in the antral follicle count, AFC), but only at a younger age^53^. Older women exhibit faster ovarian loss over time, i.e., faster reproductive aging when subjected to psychological stress at youth^53^. Earlier maturation and higher probability of conception were shown to be related to higher AFC^54,55^. Thus, this might explain the fact that in our sample women started their puberty and reproduction earlier (as having potentially higher AFC), however, ultimately did not differ in later-life parity, inter-birth intervals and end of reproductive span from women who did not experience early-life stress but also experienced ovarian loss over the life course^56^.

Our findings should be interpreted in the light of several limitations. Those include the possible memory bias as some of the reproductive data (miscarriages and age at menarche) were recalled from several decades before the participation in the study. Some previous studies documented a relatively low impact of large time interval on reproductive data recollection, e.g., Must and colleagues^57^ presented a high correlation between the reported age at menarche and the data from the original childhood records (r = 0.79, p < 0.001) in middle-aged women. A potential memory bias applies as well to the retrospective nature of the collected early-life adversities data also recalled from several decades before the assessment. Nonetheless, the challenges of quantifying childhood maltreatment apply to this area of research in general, as both retrospective and prospective approaches remain in low concordance (for a metanalysis see^58^), and both of those perspectives have their constraints^59^. Among other limitations of the study is a relatively small sample size of 105 women and lack of the sexual abuse assessment that is a part of the Childhood Trauma Questionnaire. Previous study documented a rather low incidence of sexual violence in rural, contemporary Polish school-aged children (3.2%)^60^, but there is lack of such estimations for the older generations.

Future studies replicating our results might draw from the multi-source assessment of childhood maltreatment (based on data from different sources, i.e., reports from a child and caregiver perspective) which seems to be potentially the most adequate marker of early-life adversities^59^. Additionally, body mass, body size, rate of growth, cortisol concentrations, sex hormones or markers of cellular aging assessed at youth could shed a light on potential physiological pathways and mechanisms which could mediate the observed associations. Further, since experiencing sexual abuse in childhood might contribute to adverse later-life reproductive outcomes, e.g., risky sexual behaviors^61^, or earlier pubertal onset^62,63^, upcoming studies should explore those associations, especially due to scarcity of research in the rural context.

The results of the present study could be of potential interest for public health professionals while planning screening programs and interventions (also in collaboration with healthcare professionals, social services, and mental health providers), focused on detecting and reducing the detrimental effects of early-life threats. Such interventions are crucial regarding the fact that accelerated maturation and faster reproductive history can also bring detrimental consequences to women, e.g., higher risk of breast or ovarian cancer^64,65^, or all cause and cardiovascular mortality^66^. Therefore, the tailored interventions should focus on girls experiencing adversities in early years of life, but also on adult women who already encountered accelerated puberty and earlier start of reproductive life. A promising approach, gaining increased attention recently, could be stress-reduction interventions like Mindfulness-based Stress Reduction (MBSR) techniques. The application of such strategies to reduce and/ or cope with stressful experiences was linked to improved well-being of children^67^ (but see:^68^), and decreased levels of inflammatory or biological aging markers (for a review see:^69^) which are considered as potential mediators of poor health status in adulthood. Other areas of research which might also benefit from understanding the early-life foundations of female fertility and reproductive outcomes are evolutionary demography and biological demography, especially in the light of currently observed shifts in reproductive trends worldwide.

Concluding, these findings enhance our knowledge about the link between childhood adversities and later-life reproductive strategy. It is the first study conducted in contemporary rural women with a low prevalence of birth control usage, fully traced reproductive history and with individual assessment of childhood stress and trauma-related experiences. Incorporating the potential early-life embeddings of female reproduction in future research is crucial for improving the overall and reproductive health of women, starting from the early years.

## Materials and methods

### Study group

Post-reproductive women (N = 131) born between 1929–1969 (mean age: 59.7; SD = 10.09) were recruited from the Mogielica Human Ecology Study Site. The study site consists of several villages in the rural, southern Poland^70^. The community had a very low access to the contraceptive methods for a significant period of time, and might be considered as a natural fertility population. A study by Colleran and Mace^71^ had shown that most (78%) of the 1995 randomly selected women aged 18–91, living in an area where our study was conducted, used the calendar method or no contraception at all. Almost none of the women used implants, injectables, intrauterine devices (IUDs), or diaphragms. It is worth highlighting that Polish rural areas were relatively economically underdeveloped in the mid-twentieth century with most of the households without running water, electricity, and with limited access to medical care (e.g., women usually gave birth at home with no professional assistance).

Reproductive and demographic data were collected with a personal questionnaire^72^. Study assistants were trained not to report the answers about which the participants were not certain about. Birth dates of the studied women and their children, as well as dates of marriage, were confirmed whenever it was possible in the parish records collected for other purposes^73,74^. Women also reported the number of their miscarriages and children’s deaths which enabled us to fully trace their reproductive history. Women (N = 26) who were not married or had more than one marriage, were divorced, had menopause or were widowed before the age of 45, used oral contraceptives at least once, or had at least one adopted child have been excluded from the final study group as their reproductive potential might have been limited by those factors. The final study group, therefore, included 105 women. Due to the missing data, the sample size in different models might differ. Descriptive statistics are presented in Table 3. The study was approved by the Jagiellonian University Bioethics Committee. All research was performed in accordance with relevant guidelines/regulations and written informed consent was obtained from each participant.

**Table 3:** Descriptive statistics of the study group.

	N	Mean	SD	Min	Max
Year of birth	105	1953.4	1.01	1929	1969
Education (years)	105	11.0	3.21	6.00	22.00
Age at marriage (years)	105	23.2	4.11	16.75	35.88
Age at menarche (years)	101	14.7	1.61	11.00	18.00
Age at first reproduction (years)	101	23.7	3.53	16.88	34.20
Age at last reproduction (years)	100	33.1	5.02	19.73	45.58
Number of children born	105	4.0	2.42	0	13
Number of daughters	105	1.91	1.51	0	7
Number of sons	105	2.12	1.50	0	8
Proportion of sons to daughters	101	0.5	0.26	0.00	1.00
Mean inter-birth interval (months)	97	39.0	18.96	11.59	108.00

### Reproductive and demographic data

The reproductive history data included the age at menarche, date of first (and only) marriage, the age at first and the last reproduction, number of pregnancies (including miscarriages and those that died after birth), along with children’s sex and birth dates. Proportion of sons, which reflects the relative number of sons to daughters born by each woman, was calculated as: number of sons/(number of sons + number of daughters)^75^. If a woman’s proportion of sons was “1”, it meant that all her children born were males. Mean inter-birth interval was calculated as the mean number of months between consecutive births of each woman. Information about year of birth (confirmed with use of the national identification number which includes the exact date of birth) and number of years of completed education (as proxy of socioeconomic status) was collected.

### Childhood adversity assessment

Exposure to early-life abuse and neglect (before the menarche) was assessed using the well-validated and widely used questionnaire—Childhood Trauma Questionnaire (CTQ)^76^. Experience of emotional abuse (e.g., “I thought my parents wished I had never been born”), physical abuse (e.g., “I was punished with a belt, a board, a cord, or some other hard object”), emotional neglect (e.g., “People in my family looked out for each other”- reversed question), and physical neglect (e.g., “I did not have enough to eat”) were assessed. The study population belongs to a highly religious and conservative community that has been taking part in our research for many years. We, therefore, decided not to ask questions about sexual abuse as not to breach the participants’ trust gained over the years.

The “CTQ sum” was calculated as a total value obtained within the four components (emotional and physical abuse, and emotional and physical neglect) of the questionnaire, i.e., 20 items total which was combined with the participants’ rating of each statement on a 5-point Likert scale from “Never true” to “Very often true”. The score obtained within each of the four subscales varied between 5–25, the total possible CTQ sum was 100. The mean “CTQ sum” was 29 (SD = 8.16) (Table 4). In addition, binary variables were computed for each of the four dimensions of childhood adversity (emotional abuse, physical abuse, emotional neglect, physical neglect) to classify women into those with “no exposure” vs. “any exposure” to childhood adversity. The frequencies of the presence of adversities (experiencing at least some stress) for the CTQ subscales are presented in Table 4.

**Table 4:** Frequency of the occurrence of adversities during childhoods within the “CTQ sum”, and CTQ subscales.

Child adversity variable	Mean (SD)	Frequency*
CTQ sum (potential range: 20–100)	29 (8.16)	
Emotional neglect (yes/no)		38 (36%)
Physical neglect (yes/no)		54 (51%)
Emotional abuse (yes/no)		25 (24%)
Physical abuse (yes/no)		18 (17%)

*The frequency represents how many women were exposed to at least some childhood adversity (“yes”) detected by the overall score of CTQ questionnaire or within the CTQ subscales.

### Statistical analyses

Data were analyzed with R Studio version 1.4.1106, ‘car’ and ‘pastecs’ packages^77^, and SPSS version 26.0.0.1 via General Linear Model (GLM), controlling for potential covariates. In the first step, the “CTQ sum” was entered in the analyses as a continuous variable (sum of values obtained in each category of emotional or physical abuse and neglect) to investigate the effect of the overall intensity of the experienced adversities on reproductive characteristics. Next, separate models with the binary variables for each of the four dimensions of childhood adversity (emotional abuse, physical abuse, emotional neglect, physical neglect) were computed for each of the reproductive parameters. The separate analysis was justified as the correlation coefficients between each category ranged from 0.18 to 0.64 suggesting the relatively low to moderate overlap between the subscales.

Depending on the outcome of the analyses, a combination of the following potential confounding factors were included a priori as covariates in the models: number of years of education (as a proxy of socio-economic status which might be both related to the childhood stress level and reproductive patterns^78^, year of birth (to control for secular trends in fertility and the age range of the study group), age at marriage and age at first reproduction—as in many conservative populations women are not sexually active before they are married, and the age at marriage may, therefore, impact the age at which they reproduce and their number of children^79–81^. Year of birth and number of years of education were used as covariates in all tested models. Additionally, age at first reproduction was included in the models testing the age at last reproduction, number of children and mean inter-birth interval. Age at marriage was incorporated to the models testing the age at first reproduction. Normality of the variables was assessed based on the Shapiro–Wilk test and histograms. Gamma distribution with link-log function was adopted for the skewed distributions. Poisson distribution was used in the analyses of the number of children born. There was no collinearity between the independent variables in all tested models (variance inflation factor values of the independent variables reached levels of < 1.7). There were also no interactions between the independent variables in each model. Homoscedasticity of the variances was confirmed via the Levene’s Test (all p-values > 0.05). No significant outliers were detected.

## Data Availability

The datasets generated during and/or analyzed during the current study are available from the corresponding author on reasonable request.
